# Amelioration of biased neuronal differentiation in humanized mouse model of valproic acid‐induced autism by precisely targeted transcranial magnetic stimulation

**DOI:** 10.1002/btm2.10748

**Published:** 2025-01-23

**Authors:** Yilin Hou, Youyi Zhao, Dingding Yang, Tingwei Feng, Yuqian Li, Xiang Li, Zhou'an Liu, Xiao Yan, Hui Zhang, Shengxi Wu, Xufeng Liu, Yazhou Wang

**Affiliations:** ^1^ Department of Military Medical Psychology Air Force Medical University Xi'an Shaanxi P. R. China; ^2^ Department of Neurobiology and Institute of Neurosciences, School of Basic Medicine Air Force Medical University Xi'an Shaanxi P. R. China; ^3^ State Key Laboratory of Military Stomatology & National Clinical Research Center for Oral Diseases & Shaanxi Engineering Research, Center for Dental Materials and Advanced Manufacture, Department of Anesthesiology, School of Stomatology Air Force Medical University Xi'an Shaanxi P. R. China; ^4^ Key Laboratory of Biomedical Information and Engineering, School of Life Sciences and Technology, Ministry of Education Xi'an Jiaotong University Xi'an Shaanxi P. R. China; ^5^ Shaanxi Brain Modulation and Scientific Research Center Xi'an Shaanxi P. R. China; ^6^ School of Management Xi'an Jiaotong University Xi'an Shaanxi P. R. China

**Keywords:** autism spectrum disorder, humanized mouse, precisely targeted TMS, valproic acid

## Abstract

Autism spectrum disorder (ASD) is a group of developmental diseases, which still lacks effective treatments. Pregnant exposure of Valproic acid (VPA) is an important environmental risk factor for ASD, but it's long‐term effects on the development of human neural cells, particularly in vivo, and the corresponding treatment have yet been fully investigated. In the present study, we first made a humanized ASD mouse model by transplanting VPA‐pretreated human neural progenitor cells (hNPCs) into the cortex of immune‐deficient mice. In comparison with wild type and control chimeric mice, ASD chimeric mice (^VPA^hNPC mice) exhibit core syndromes of ASD, namely dramatic reduction of sociability, social interaction and social communication, and remarkable increase of stereotype repetitive behaviors and anxiety‐like behaviors. At cellular level, VPA‐pretreatment biased the differentiation of human excitatory neurons and their axonal projections in host brain. Chemogenetic suppression of human neuronal activity restored most behavior abnormalities of ^VPA^hNPC mice. Further, specific modulation of human neurons by a newly developed transcranial magnetic stimulation (TMS) device which could precisely target hPNCs effectively recued the core syndromes of ASD‐like behaviors, restored the excitatory‐inhibitory neuronal differentiation and axonal projection, and reversed the expression of over half of the VPA‐affected genes. These data demonstrated that ^VPA^hNPC mice could be used as a humanized model of ASD and that precisely targeted TMS could ameliorate the VPA‐biased human neuronal differentiation in vivo.

**Translational Impact Statement**

The humanized ASD mouse model offers a platform for disclosing human‐specific mechanism of ASD and evaluating new treatments by directly investigating human ASD neurons. Precisely targeted TMS could be applied to ASD patients by specifically modulating key brain regions.

## 1 INTRODUCTION

Autism spectrum disorder (ASD) is one of the most prevalent developmental diseases featured by social defects and stereotype repetitive behaviors. General views hold that ASD is caused by both genetic and environmental risk factors. So far, over 1000 risk genes have been identified and extensive mechanistic studies have been carried out.^1^ Multiple hypothesis, including “imbalance of excitatory / inhibitory synaptic transmission”,^2^ “mitochondrial dysfunction”^3^ and “over‐pruning of synapses^4^”, have been proposed as the potential mechanisms of ASD development. Multiple therapeutic targets, such as Wnt/mTOR/PI3K signaling,^5^ gut microbes and glutamate receptors,^6^, ^7^ have been identified and are under investigation. However, there still lacks effective therapy in clinic. Current treatments are mostly behavior oriented, often unsatisfying in alleviating core syndromes.^8^, ^9^ One important reason for the low efficiency of translating animal studies into clinic lies in the species difference between mouse models and human patients.

Relative to genetic risk factors, how environmental factors induce ASD remains poorly investigated.^10^, ^11^ Valproic acid (VPA), an anti‐epilepsy drug used to be prescribed for pregnant woman, has been widely adopted as representative environmental risk factor to model ASD in rodents.^12^ Animal studies have revealed that utero exposure of VPA affected neural development by aberrantly increasing Wnt/β‐catenin signaling and the ratio of excitation/inhibition of synaptic transmission.^13^, ^14^ How VPA influences human neural cells, particularly in vivo, are largely unclear.

Recent progresses in human neural cell chimeric mice provided a new platform for probing human‐specific neuropathological mechanisms. By adopting human microglia chimeric mice, researchers have examined the in vivo function of patient‐derived microglia and discovered type‐I‐interferon signaling as a new mechanism for Alzheimer's disease and Down syndrome.^15^ By using human glial progenitor chimeric mice, researchers revealed the importance of astrocytes in JC virus infection.^16^ In human neuron chimeric mice, human neurons show species‐specific development and functional integration into mouse circuits,^17^ opening a door of directly investigating the behaviors of human neurons in vivo. Therefore, studies on humanized ASD mouse would be helpful for uncovering unnoticed mechanisms and testing potential therapeutic methods.

In the present study, we established a humanized ASD mouse model by transplanting VPA‐pretreated human neural progenitor cells (hNPCs) into the cerebral cortex of immunodeficient mice and examined the in vivo development of human neurons. Then, we assessed the therapeutic potential and the underlying mechanism of a newly developed transcranial stimulation (TMS) device which could precisely modulate neuronal activities of hNPC graft.

## 2 MATERIALS AND METHODS

### 2.1 Animals and ethic statements

Adult NOD‐SCID mice were bought from the animal center of the Fourth Military Medical University. All animal experiments were carried out under protocols approved by the Animal Care and Use Committee of Fourth Military Medical University (KY20213361‐1) and according to the ARRIVE guidlines 2.0. All the experiments of human neuronal chimeric mice were conducted under protocols approved by the ethic comittee of medical research of Fourth Military Medical University. For all experiments, animals were simultaneously randomized to the treatment groups without considering any other variable.

### 2.2 Human embryonic stem cell culture and neuronal induction

Human embryonic stem cell line H8 (Sex: male. RRID:CVCL_B207) was obtained from Prof. Wei Jiang (Wuhan University). Cells were maintained by mTeSR1 medium (Cat. 85850, STEMCELL Technologies, Canada). Neural induction was conducted as reported with minor modification.^18^ Briefly, hESCs were digested using accutase and cultured using neural induction medium (NIM, Cat. 08582, STEMCELL Technologies, Canada) for 7 days. After embryonic body (EB) formation, EBs were transferred to 6‐well plates pre‐coated with poly‐D‐lysine and cultured until rosette formation. Rosettes were digested by neural rosette selection reagent (Cat#05832, STEMCELL Technologies, Canada), and cultured with 50% neuron‐differentiation medium (NDM) and 50% neurobasal containing 2% B27, 100 nmol/L brain‐derived neurotrophic factor (BDNF, Cat. 45002, Peprotech) and 100 nmol/L glia‐derived neurotrophic factor (GDNF, Cat. 45010, Peprotech) until robust neuron generation.

### 2.3 VPA‐treated hNPC chimeric mouse

Humanized neuronal chimeric mice were established as described.^19^ Briefly, selected rosette cells were treated with VPA (1 mM, P4543, Sigma) or vehicle solution for 3 days as described.^20^ Then, VPA‐pretreated hNPCs were digested and suspended in culture medium containing 5 mM EGTA as suggested.^17^ 1–2 × 10^5^ cells were transplanted into the bilateral somatosensory cortex of immunodeficient NOD‐SCID pups (P0‐P2) to make chimeric mouse. At 1 or 2 months after transplantation, chimeric mice were used for experiments. As the sex of hNPCs is male, male chimeric mice were adopted for experiments to minimize the possible sex‐based discrepancies.

### 2.4 Chemogenetic manipulation in VPA‐pretreated hNPC chimeric mice

Human embryonic stem cell line H8 was transfected with LV‐CaMKII‐hM4Di‐mCherry‐WPRE (OBIO Technology Corp., Ltd. Shanghai). mCherry‐positive cells were separated by flow cytometric cell sorting and expanded. Subsequently, mCherry‐positive H8 cells were induced towards neuronal fate and treated by VPA as above mentioned. Subsequently, the cells were collected for transplantation. When chimeric mice were 2 month‐old, 1 mg/kg clozapine‐N‐oxide (CNO) (RTI International; North Carolina, United States) was injected for 5 consecutive days. Then behavior analysis was conducted.

### 2.5 Immunohisto(cyto)chemistry and TdT‐mediated dUTP nick end labeling staining

For immunohistochemistry, mice were perfused intracardially with 4% paraformaldehyde phosphate buffer. Serial coronal sections were prepared and blocked by PBS containing 3% BSA and 0.3% Triton‐X100, and then incubated with primary antibodies overnight at room temperature.

For immunocytochemistry, cells were fixed with 4% paraformaldehyde (PFA) for 30 min at room temperature, and then permeabilized with 0.1% triton‐X100 for 10 min. The primary antibodies used were as the following: mouse anti‐Nestin (GeneTex, GTX630201, RRID:AB_2888203, 1:200), rabbit anti‐Ki67 (GeneTex, GTX16667, RRID:AB_422351, 1:200), goat anti‐GFP (GeneTex, GTX26673, RRID:AB_371426, 1:400), mouse anti‐Tuj‐1 (abcam, ab78078, RRID:AB_2256751, 1:100), rabbit anti‐CaMK II (GeneTex, GTX135117, RRID:AB_2887447, 1:400), rabbit anti‐VGLUT1 (GeneTex, GTX133148, RRID:AB_2801548, 1:500), rabbit anti‐PSD95 (abcam, ab18258, RRID:AB_444362, 1:200), rabbit anti‐c‐Fos (Cell Signaling Technology, #2250, RRID:AB_2247211, 1:500). After washing with PBS, corresponding secondary antibodies conjugated with donkey anti‐rabbit (Alexa Fluor 594, Invitrogen, A‐21207 RRID:AB_141637, 1:800), donkey anti‐mouse (Alexa Fluor 594, Invitrogen, A‐21203, RRID:AB_141633, 1:800), donkey anti‐goat (Alexa Fluor 488, Invitrogen, A‐11055, RRID:AB_2534102, 1:800), were incubated with the sections for 2–4 h at room temperature protected from light. After washing with PBS, sections were counterstained with Hoechst33342 (1:1000, Sigma) for 20 min.

For TdT‐mediated dUTP nick end labeling (TUNEL) staining, cells were fixed by 4% PFA and washed by PBS. Then cells were incubated with 0.3% Triton X100 in PBS and subsequently with TUNEL staining solution for 60 min at 37 according to the manual of commercial kit (G3250, Promega).

### 2.6 Western‐blotting

hNPC grafts were carefully dissected and lysed by RIPA buffer at the presence of proteinase inhibitor cocktails. Protein concentration was determined by bicinchoninic acid assay. Protein samples were separated in 10%–12% acrylamide gels by sodium dodecyl sulfate–polyacrylamide gel electrophoresis and transferred to polyvinylidene fluoride membranes. Membranes were blocked in TBS containing 0.1% (v/v) Tween 20 and 5% (w/v) nonfat milk before incubating with primary antibodies. The following antibodies were used: mouse anti‐Tuj‐1 (abcam, ab78078, RRID:AB_2256751, 1:1000), rabbit anti‐CamKII (GeneTex, GTX135117, RRID:AB_2887447, 1:1000), rabbit anti‐VGLUT1 (GeneTex, GTX133148, RRID:AB_2801548, 1:1000), mouse anti‐GAD67(Millipore, MAB5406, RRID:AB_2278725, 1:1000), rabbit anti‐SYP (abcam, ab32127, RRID:AB_2286949, 1:1000). After washes, membranes were incubated with HRP‐conjugated anti‐mouse secondary antibody (Cat. CW0102S, 1:3000, CWBIO Co., Ltd), or HRP‐conjugated anti‐rabbit secondary antibody (Cat. EK020; 1:3000; Zhuangzhi Biotech Co., Ltd). Bands were visualized by an ECL kit (Zeta life, 310231). Images were analyzed by Image J. For quantification of blots, the ratios of (gray scale of target protein)/(gray scale of β‐actin) in experimental groups were compared to those of control groups.

### 2.7 Behavior tests

#### 2.7.1 Three‐chamber test

The 3‐chamber apparatus was made by an opaque acrylic box (65 × 45 × 25 cm) with three chambers (43 × 23 cm). After habituation, a stimulus mouse was placed in the cylinder in the ‘social chamber’, with the cylinder in the ‘non‐social chamber’ remaining empty. The time that test mice spent in the social versus non‐social chambers was measured. In social novelty test, a novel mouse was put into the non‐social chamber. The time the test mice spent in the chamber with familiar mouse versus the chamber with novel mouse was measured. The behavior of each mouse was video‐recorded. Each chamber was cleaned with 75% ethanol between tests. The behavior was analyzed using SMART3·0 software (Panlab Harvard Apparatus, Spain). The (Time social − Time non‐social)/(Time social + Time non‐social) was calculated as preference score.

#### 2.7.2 Resident‐juvenile‐intruder test

The test was performed as previously described. Briefly, the resident mouse (test mouse) was allowed to explore freely in its home cage. An intruder mouse (novel, 3–4 weeks old) was put into the resident cage. Juvenile intruder was used to avoid mutual aggression. The test mouse was allowed to explore the intruder mouse freely for 10 min. The time and frequency of direct contacts were measured.

#### 2.7.3 Open field test

The open field test was carried out in a white opaque plastic chamber (50 × 50 × 35 cm) as described^21^ with minor modification. The open field was divided into 25 squares with same area. The central nine squares in were defined as central area, and the remaining as periphery area. For each test, mouse was gently placed in one corner, and the movement was recorded for 5 min with a video tracking system. The time spent and distance traveled in the central area and the total distance traveled in the field were measured using the SMART software (SMART 3.0, Panlab S.L.U.). Between each test, 75% ethanol was used to clean the open field area.

#### 2.7.4 Elevated cross maze test

The maze was placed 50 cm above the floor and consisted of two open arms and two closed arms (30 × 5 cm and 15 cm wall height for the closed arms). Each mouse was placed onto the center area, heading towards the same open arm, and videotaped in the following 5 min. The time spent and moving distance in the open arms, and the total movements in both open and closed arms were analyzed using the software SMART 3.0. The maze was cleaned by 75% ethanol between tests.

#### 2.7.5 Ultrasonic vocalization recording

Ultrasonic vocalization (USV)recordings were performed as described. Briefly, mice were placed in clean rectangular cage (60 × 42 × 40 cm) covered by a metal lid. A field microphone (VT UltraMic‐384, Virtins Technology) was placed 2 cm below the lid in the center of the cage. The microphone signal was sampled by software (SeaWave) recording the frequency ranging from 20 Hz to190 kHz. One male testing mouse and one female wild type (WT) mouse were put in the USV recording cage. Each group of mice had no interactions prior to the recording and was considered as *n* = 1 for statistics. USV production was recorded for 10 min. Data were stored and analyzed off line by a researcher blind to experimental design. USV was detected by a custom program (Deep squeak) with a spectrographic display.

#### 2.7.6 Grooming test

The grooming test was performed as described. For each test, the tested mouse was put into a cage (20 cm × 20 cm × 25 cm) with three opaque walls, one clear wall, and an opaque bottom, and video recorded for 30 min. The number of grooming sessions, total grooming time and latency time of the first session were analyzed by an experienced researcher blind to the treatment.

#### 2.7.7 Marbles burying test

Before testing, a cage with bottom covered by 5 cm wood chip bedding was prepared. Mice were put into the cage for habituation for 20 min. Then, 12 glass marbles were evenly placed on the surface of bedding with 4 cm apart from each other. Then, tested mouse was placed in the cage, and video was recorded for 20 min. At the end of experiments, the video was analyzed and the number of marbles (buried or not) was counted.

#### 2.7.8 Fear conditioned memory

All mice were acclimated in the test chamber for 5 min 1 day before training. During training period, mice were placed in the experimental chamber for 3 min before being given 30 s of sound stimulation, 2 s of electrical stimulation, and 1 min of no stimulation, for a total of 5 training sessions. After 24 h, mice were allowed to move freely in the test chamber for 3 min, with 30 s of sound stimulation. The mice's rigidity time was recorded after 1 min of no stimualtion. After 24 h, the mice were placed into a new test chamber of different shape and allowed to move freely for 3 min and then subjected to 30 s of sound stimulation. After 1 min, mice's rigidity time was recorded.

#### 2.7.9 Novel object recognition test

On the first day, the test mouse was placed in open field for 10 min of free exploration. On the second day, the test mouse placed in the same open field with two identical building blocks and allowed to explore for 10 min. On the third day, a block was replaced by a novel block of a different shape, and the mouse was allowed for 10 min of exploration. The exploration preference of mice for new and old building blocks was calculated.

### 2.8 Precisely targeted TMS treatment

#### 2.8.1 Brain region specificity test of precisely targeted TMS

TMS device equipped with a 13 layer 3‐turn “8”‐shape coil (Black Dolphin IT‐TMS, Solide Company, Xi'an, China) was adopted. Brain region specificity was tested by stimulating the hindlimb region of right motor cortex. Upon single TMS stimulation, movement of bilateral hindlimbs was videoed. At the same time, electromyographic activity in bilateral hindlimbs was recorded. Briefly, the positive electrode was inserted into the front end of the gastrocnemius muscle and the negative electrode the back end of the gastrocnemius muscle. The single channel signal sampling rate was set at 8000 Hz with the highest rate of 32,000 Hz. The signal was filtered through a 50 Hz band‐stopping filter, a 20–480 Hz band‐passing filter, and a power frequency harmonic filter (Solide Company, Xi'an, China).

#### 2.8.2 Precisely targeted TMS treatment

The coil was placed 1–2 mm above the skull where hNPC transplantation was made. The parameters of rTMS were as follows: 1 Hz, stimulating pulse intensity at 40% of the maximum powe, 10 stimulations per cluster, repeated 60 times. rTMS was conducted for 7 consecutive days, with 600 pulses per day. Starting from 3 days prior to the rTMS procedure, the mice were habituated to the coil for 10 min each day. For sham stimulation, the coil was placed immediately above the skull without magnetic stimulation. For electric field simulation, the open accessed electromagnetic simulation software (Simnibs) was used as described,^22^, ^23^ and the current change rate was set at approximately 40% of the maximum current change rate.

### 2.9 RNA‐seq and qPCR

For RNA‐seq, total RNA was isolated from hNPC graft in control chimeric mice, ^VPA^hNPC mice, or rTMS‐treated ^VPA^hNPC mice using Trizol reagent kit (Invitrogen, Carlsbad, CA, USA) according to the manufacturer's protocol. RNA quality assessment, reverse transcription, PCR amplification and sequencing, and data analysis were performed by Gene Denovo Biotechnology Co. (Guangzhou, China).

For real‐time quantitive RT‐PCR (qPCR), total RNA was extracted as above. After reverse transcription, cDNA was mixed with qPCR buffer and amplified using a standard protocol (95°C 1 min for initial degeneration, 95°C 5 s, 60°C 20 s cycles for amplification). The primers used were as the following:

*LZTS1‐F*: AGCGTCAGTAGCCTCATCTC

*LZTS1‐R*: AGTCTTCGCTCTTGCCCATTT

*WNT2‐F*: CCGAGGTCAACTCTTCATGGT

*WNT2‐R*: CCTGGCACATTATCGCACAT

*TNC‐F*: TCCCAGTGTTCGGTGGATCT

*TNC‐R*: TTGATGCGATGTGTGAAGACA

*HAND2*‐F: ATGAGTCTGGTAGGTGGTTTTCC

*HAND2*‐R: CATACTCGGGGCTGTAGGACA

*SCRT2*‐F: GCAAGACCTACGCCACGTC

*SCRT2*‐R: CAGCGGTAGTGCTTGAAGG

*SCRT1‐F*: CCTCGTCCGTCTACGATGG

*SCRT1‐R*: GCCCGTCGGTGATGAAGAA

### 2.10 Statistical analysis

All behavior analysis and statistics were performed by an investigator who was blinded to experimental design. No sample calculation was performed. For in‐vitro study, 3–5 batches of cells were used for each experiment. For in‐vivo study, at least 3 mice were included in each group for morphological analysis, and 8 mice were included in each group for behavior analysis. Each behavior test was conducted using distinct groups of animals. Data were presented as the mean ± standard error. Normally distribution was assessed by Shapiro–Wilk test. Statistical comparisons were made using Student's *t*‐test or one‐way ANOVA with Student Newman–Keuls post hoc analysis. *p* Value less than 0.05 was considered as statistically significant.

## 3 RESULTS

### 3.1 Establishment of VPA‐induced humanized ASD mice

**FIGURE 1:**
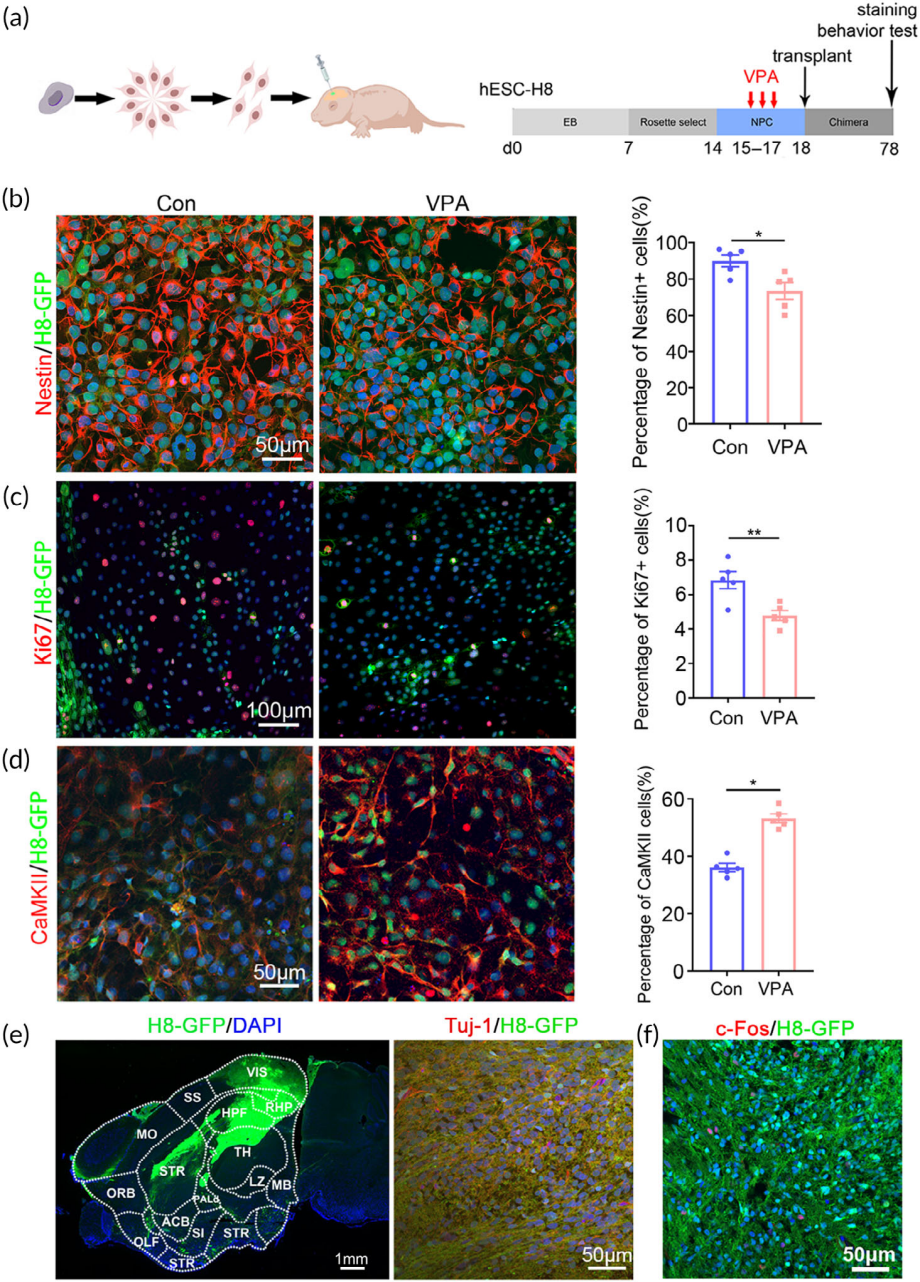
Establishment of VPA‐pretreated human neuron chimeric mice (^VPA^hNPC mice). (a) Experimental design for neural induction, VPA treatment and cell transplantation. (b) Double‐immunostaining and quantification of Nestin/H8‐GFP in hNPCs 3 days following VPA treatment. (c) Double‐immunostaining and quantification of Ki67/H8‐GFP in hNPCs 3 days following VPA treatment. (d) Double‐immunostaining and quantification of CaMKII/H8‐GFP in hNPCs 3 days following VPA treatment. (e) Immunostaining of GFP in the sagittal sections of ^VPA^hNPC mice (left panel) and double‐immunostaining of Tuj‐1/H8‐GFP in ^VPA^hNPC mice (right panel). (f) Double‐immunostaining of c‐Fos/H8‐GFP in ^VPA^hNPC mice following social stimulation. Notice the wide distribution of GFP‐positive human nerve fibers in mouse brain and the expression of c‐Fos in human neurons. *N* = 5 batches of cells in (b–d). Students' *t* test in “b–d”. *p < 0.05.**p < 0.01. Con, control.

To establish a VPA‐induced humanized ASD mouse model, we adopted human embryonic stem cell line H8 (hESC‐H8) which stably expressed GFP, and induced hESC‐H8 towards hNPCs for transplantation. VPA was added to the culture medium of hNPCs at 3 days following rosette formation when approximately 90% cells expressed Nestin (the marker of neural stem cells, Figure 1a; Figure S1A). Following VPA treatment, the expression of Nestin and Ki67 (marker of cell proliferation) significantly decreased as compared with vehicle control (Figure 1b,c). In the meanwhile, the expression of Tuj‐1 and CamKII (markers of pan‐neurons and excitatory neurons) increased remarkably (Figure 1d; Figure S1B). To assess if VPA treatment was toxic to hNPCs, we performed TUNEL staining. Very rare TUNEL‐positive cells were detected in both control and VPA‐treated hNPCs (Figure S1C), indicating that VPA did not induce apoptosis of hNPCs. These data illustrated a differentiation‐inducing and proliferation‐inhibiting effects of VPA on hNPCs.

Then, hNPCs treated with or without VPA were transplanted into the bilateral somatosensory cortex of neonatal NOD‐SCID mice (P0‐P2). Grafted human neural cells survived well in mouse brain. Two‐month after transplantation, GFP‐positive human axons and terminals were observed in most brain regions, including olfactory bulb, cerebral cortex, striatum, hippocampus and thalamus (Figure 1e, left panel). Co‐localization of Tuj‐1/GFP confirmed the neuronal differentiation of hNPCs in mouse brain (Figure 1e, right panel). Exposing human neuron chimeric mice to social stimulation induced rapid appearance of c‐Fos in human neurons (Figure 1f), indicating the integration and function of human neurons in mouse brain.

**FIGURE 2:**
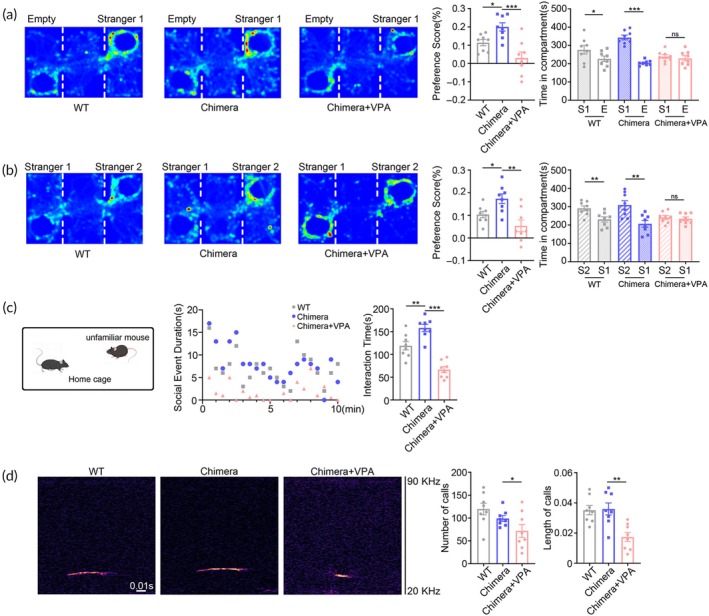
Social behaviors of ^VPA^hNPC mice. (a) Three‐chamber tests of social preference. (b) Three‐chamber tests of social novelty. Notice the decrease of social preference and social novelty in ^VPA^hNPC mice. (c) Resident‐intruder assay. (d) Ultrasonic vocalization. Notice the decrease of social interaction and ultrasonic communication in ^VPA^hNPC mice. *N* = 8 mice per group. One way ANOVA. *p < 0.05.**p < 0.01. ***p < 0.001. WT, wild type. Chimera, control hNPC chimeric mice. Chimera+VPA, ^VPA^hNPC mice.

We next examined whether the chimeric mice made from VPA‐pretreated hNPCs (^VPA^hNPC mice) exhibited ASD‐like behaviors by comparing them with control chimeric mice (made from normal hNPCs) and WT mice. To assess the social function of humanized mice, we conducted three‐chamber test, resident‐intruder assay and ultrasonic vocalization recording. In the social preference stage of three‐chamber test, ^VPA^hNPC mice spent similar time exploring the social and non‐social chambers while control chimeric mice and WT mice stayed longer in the social chamber (Figure 2a), suggesting a reduction of social preference. In the social novelty stage of three‐chamber test, ^VPA^hNPC mice spent similar time in the chamber having familiar mouse and the chamber having novel mouse (Figure 2b), suggesting a loss of interest in social novelty. In resident‐intruder assay which assess animal's sociability, ^VPA^hNPC mice spent significantly less time interacting with the intruder mice during the session of test, as compared with that of control chimeric mice and WT mice (Figure 2c). Further, we examined the social communication of ^VPA^hNPC mice by recoding the animals' ultrasonic vocalization. Dramatic reduction of ultrasonic emissions, both in number and duration, was detected in ^VPA^hNPC mice, in comparison with that in control chimeric mice and WT mice (Figure 2d).

**FIGURE 3:**
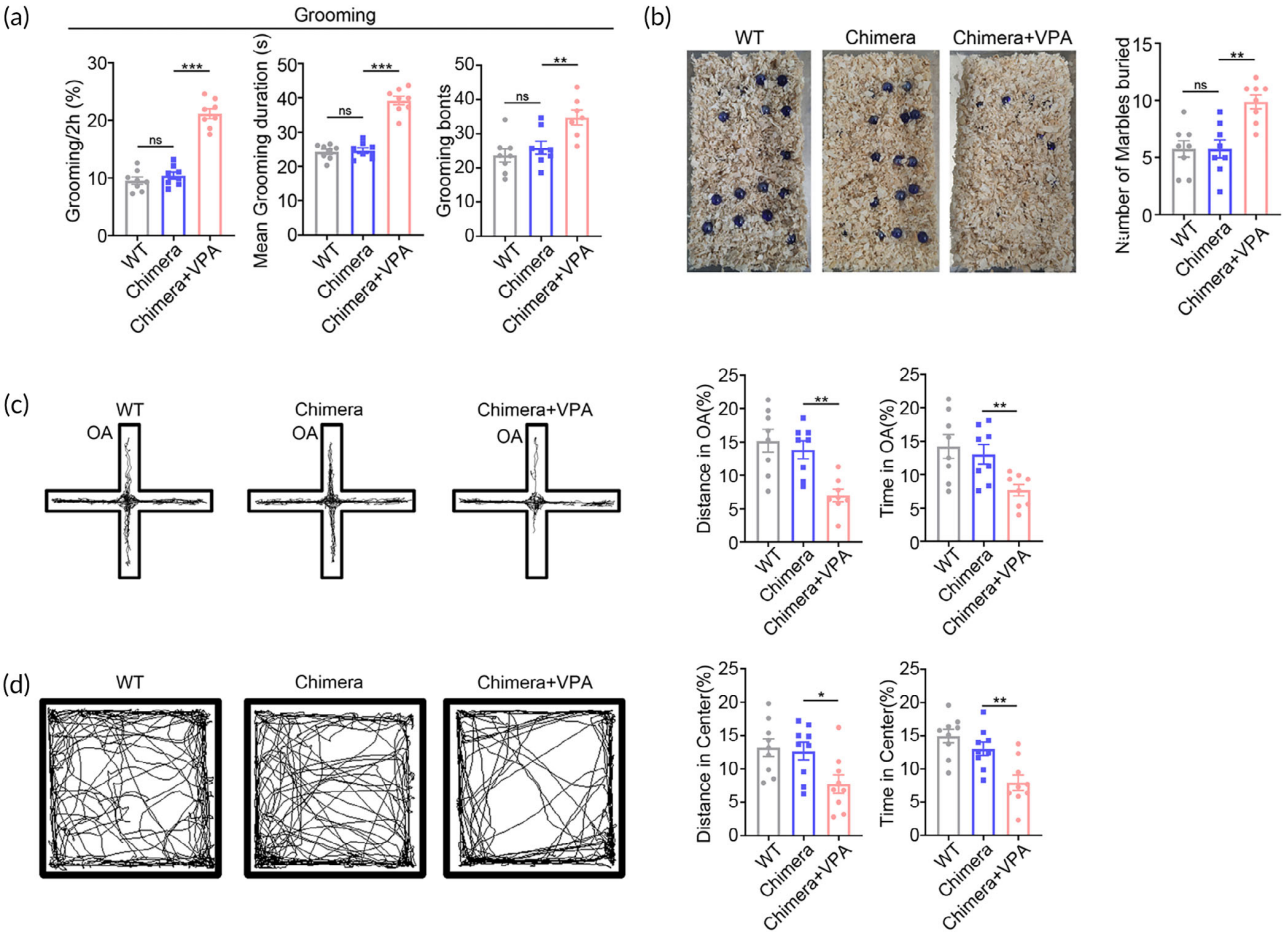
Repetitive and anxiety‐like behaviors of ^VPA^hNPC mice. (a) Grooming test of WT mice, control chimeric mice and ^VPA^hNPC mice. (b) Marble burying test. Notice the increase of grooming and marbles buried in ^VPA^hNPC mice. (c) Elevated plus maze test of WT mice, control chimeric mice and ^VPA^hNPC mice. (d) Open field test of WT mice, control chimeric mice and ^VPA^hNPC mice. ^VPA^hNPC mice showed reduced movement in the open arm and center field. *N* = 8 mice per group. One way ANOVA. *p < 0.05.**p < 0.01. Chimera, control chimeric mice; VPA, ^VPA^hNPC mice; WT, wild type.

In grooming test which evaluates animals' repetitive behavior, ^VPA^hNPC mice spent significantly longer time self‐grooming as evidenced by longer mean grooming duration and more grooming bouts, than that of both control chimeric mice and WT mice (Figure 3a). In marble burying test which also reflects repetitive behaviors, ^VPA^hNPC mice buried more marbles than control chimeric mice and WT mice did (Figure 3b). In both tests, control chimeric mice showed similar self‐grooming behavior and marble burying activity with WT mice (Figure 3a,b), suggesting that the repetitive behavior was ^VPA^hNPC mice specific.

As ASD patients are usually burdened by comorbid psychiatric disorders,^24^ we next explored if ^VPA^hNPC mice exhibit anxiety‐like behaviors or cognition decline. In both open field and elevated cross maze tests, ^VPA^hNPC mice showed significantly less movement in the center area of open field and open arm (OA) of the elevated plus maze, as compared with control chimeric mice and WT mice (Figure 3c,d), indicating a high level of anxiety. In fear memory test and novel object recognition test, ^VPA^hNPC mice exhibited similar behavior as that of control chimeric mice (Figure S2A,B). Interestingly, in most above tests, control chimeric mice showed similar behaviors with WT mice (Figure 3). Taken together, these data demonstrated that, in comparison with WT and control chimeric mice, ^VPA^hNPC mice displayed the core phenotypes of ASD, and could be used as a humanized ASD mouse model.

### 3.2 Biased differentiation and axonal projection of human neurons in ^VPA^hNPC mice

**FIGURE 4:**
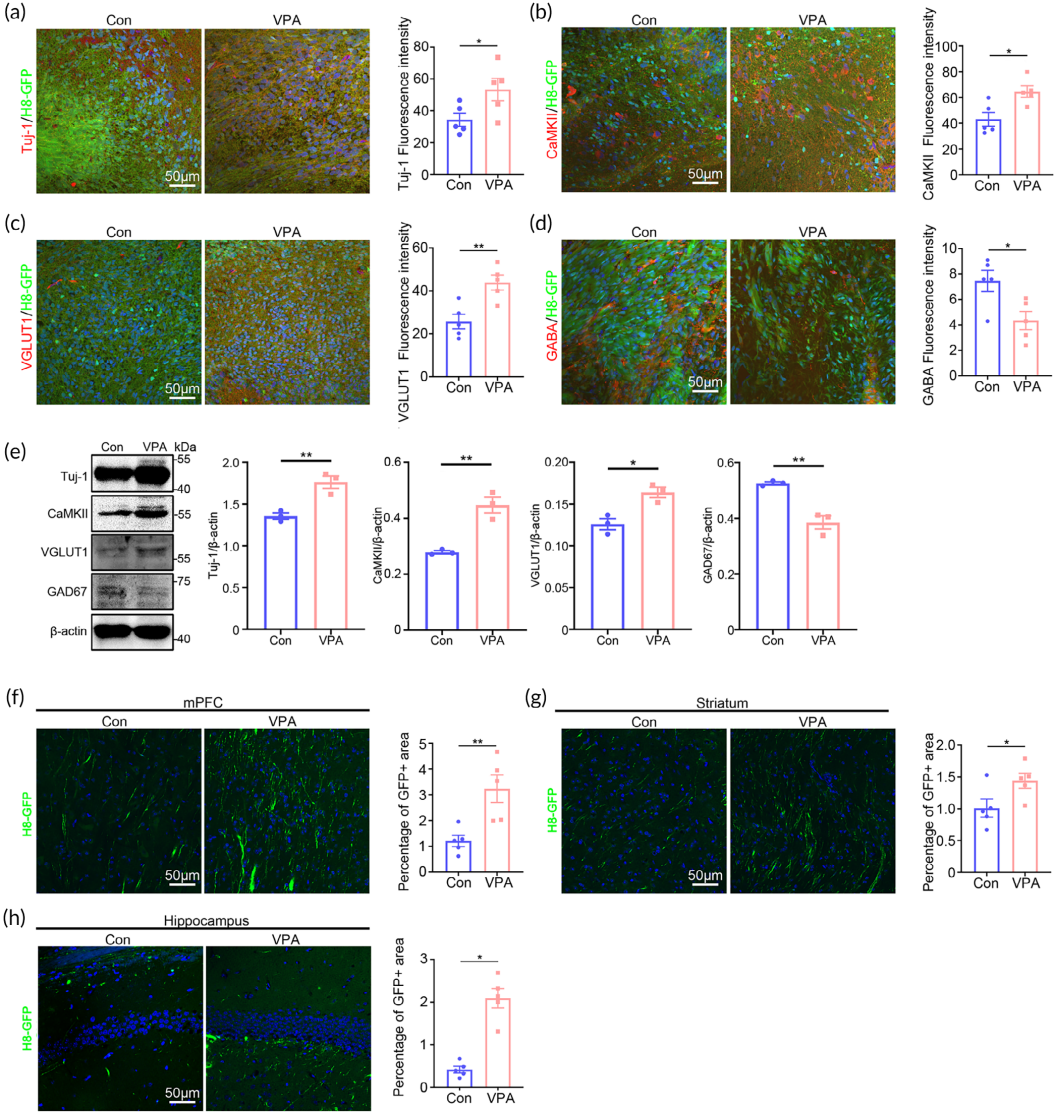
Biased excitatory neuronal differentiation and axonal projection of human neurons in ^VPA^hNPC mice. (a) Double‐immunostaining of Tuj1/H8‐GFP in control chimeric mice and ^VPA^hNPC mice, and quantification. (b) Double‐immunostaining of CaMKII/H8‐GFP in control chimeric mice and ^VPA^hNPC mice, and quantification. (c) Double‐immunostaining of Vglut1/H8‐GFP in control chimeric mice and ^VPA^hNPC mice, and quantification. (d) Double‐immunostaining of GABA/H8‐GFP in control chimeric mice and ^VPA^hNPC mice, and quantification. Notice the enhanced generation of CaMKII‐positive and Vglut1‐positive neurons and decreased generation of GABA‐positive neurons from VPA‐pretreated hNPCs. (e) Western‐blotting and quantification of Tuj‐1, CamKII, vGlut1 and GAD‐67 in the hNPC graft of control chimeric mice and ^VPA^hNPC mice. (f–h) GFP‐positive human axons in the mPFC, striatum and hippocampus of control chimeric mice and ^VPA^hNPC mice. Notice that there were more human axons in the mPFC, striatum and hippocampus of ^VPA^hNPC mice. *N* = 5 mice per group. Student's *t* test. *p < 0.05. **p < 0.01. Con, control chimeric mice; VPA, ^VPA^hNPC mice.

As control chimeric mice showed similar behaviors with WT mice in most behavior tests, we explored how VPA‐pretreated hNPC induced ASD‐like behaviors by comparing the neuronal differentiation and axon growth between ^VPA^hNPC mice and control chimeric mice. Double‐immunostaining of pan‐neuron marker (Tuj‐1), excitatory neuron markers (CaMKII, vGlut1) and inhibitory neuron marker (GABA) with GFP showed significantly more Tuj‐1/GFP‐, CamKII/GFP‐ and vGlut1/GFP‐positive cells, but less GABA/GFP‐positive cells in the ^VPA^hNPC mice, as compared with that of control chimeric mice (Figure 4a–d). Further Western‐blotting confirmed the higher levels of Tuj‐1, CamKII and vGlut1 protein and lower levels of GAD67 in the hNPC graft of ^VPA^hNPC mice, as compared with that of control chimeric mice (Figure 4e). In terms of synaptogenesis, immunohistochemistry and Western‐blotting showed that VPA pretreatment had no significant effects on the expression of PSD‐95 and Synaptophysin in hNPC grafts (Figure S3A,B). These data indicated that VPA‐pretreatment favored the differentiation of hNPCs towards excitatory neurons.

Next, we analyzed the axonal projections of human neurons in the key brain regions which were closely associated with ASD syndrome. Significantly more human axons were found in the medial prefrontal cortex (mPFC), striatum and hippocampus of ^VPA^hNPC mice, as compared with the corresponding regions of control chimeric mice (Figure 4f–h). These data indicated that over innervation of these brain regions by VPA‐pretreated human neurons might contribute to the ASD‐like behaviors of ^VPA^hNPC mice. As the projection of human axons in other brain regions (such as olfactory bulb and thalamus) are relatively sparse in both control and ^VPA^hNPC mice, we did not perform quantification.

### 3.3 Chemogenetic inhibition of human neurons ameliorates ASD‐like behaviors of ^VPA^hNPC mice

**FIGURE 5:**
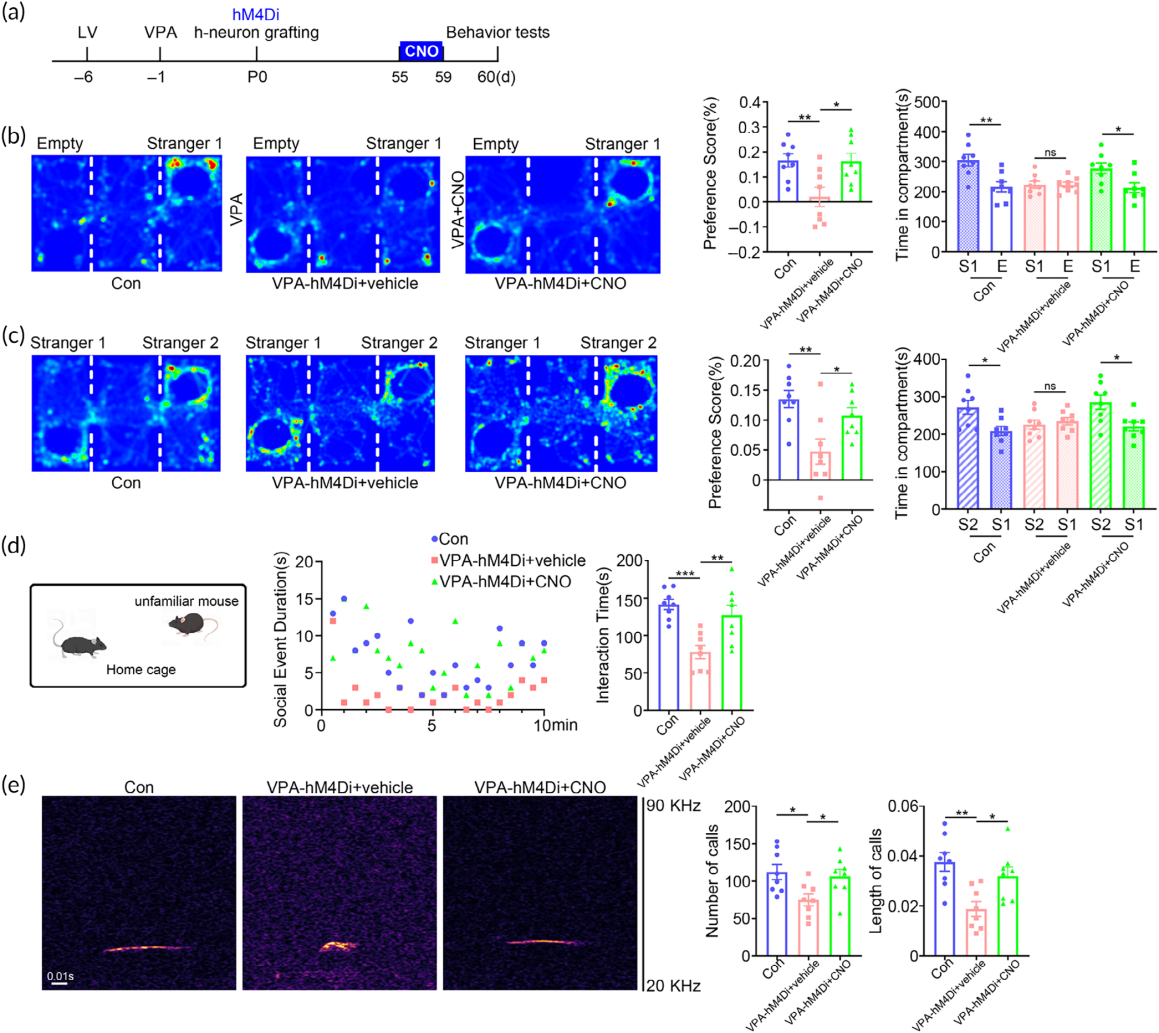
Alleviation of social defects of ^VPA^hNPC mice by chemogenetically inhibiting human excitatory neurons. (a) Experimental design for “b–e”. (b, c) 3‐chamber test of WT mice, ^VPA‐hM4Di^hNPC mice treated with vehicle or CNO. (d) Resident‐intruder assay of WT mice, ^VPA‐hM4Di^hNPC mice treated with vehicle or CNO. (e) Ultrasonic vocalization of WT mice, ^VPA‐hM4Di^hNPC mice treated with vehicle or CNO. Notice the improvement of social preference, social interaction and social communication of ^VPA‐hM4Di^hNPC mice by CNO. *N* = 8 mice per group. One way ANOVA in “b–e”. *p < 0.05.**p < 0.01. ***p < 0.001. CNO, clozapine‐N‐oxide; Con, control chimeric mice; VPA‐hM4Di, ^VPA‐hM4Di^hNPC mice.

Above data indicated that the relative high levels of excitatory neuronal activities of human neurons might lead to the ASD‐like behaviors of ^VPA^hNPC mice. To test the function VPA‐pretreated human neurons, we performed chemogenetic manipulation. Considering that VPA‐pretreated hNPCs generated more excitatory neurons, we transfected hESC‐H8 cells with lentivirus expressing inhibitory hM4Di DREADD under the control of excitatory neuron specific promoter. hM4Di‐infected hESCs were induced to hNPCs, treated with VPA, and then transplanted to make chimeric mice (^VPA‐hM4Di^hNPC mice). At 2‐month, CNO was administered for 5 successive days and mice behaviors were evaluated subsequently (Figure 5a). Upon CNO treatment, ^VPA‐hM4Di^hNPC mice exhibited robust increase of social preference in 3‐chamber test and enhancement of social interactions in resident‐intruder assay (Figure 5b,c). In male–female ultrasonic vocalization recording, CNO treated ^VPA‐hM4Di^hNPC mice emitted much more voices than vehicle treated mice. In addition, the average length of ultrasonic voice was much longer in CNO treated ^VPA‐hM4Di^hNPC mice, as compared with that of vehicle treated mice (Figure 5e), indicating the recovery of social communication.

**FIGURE 6:**
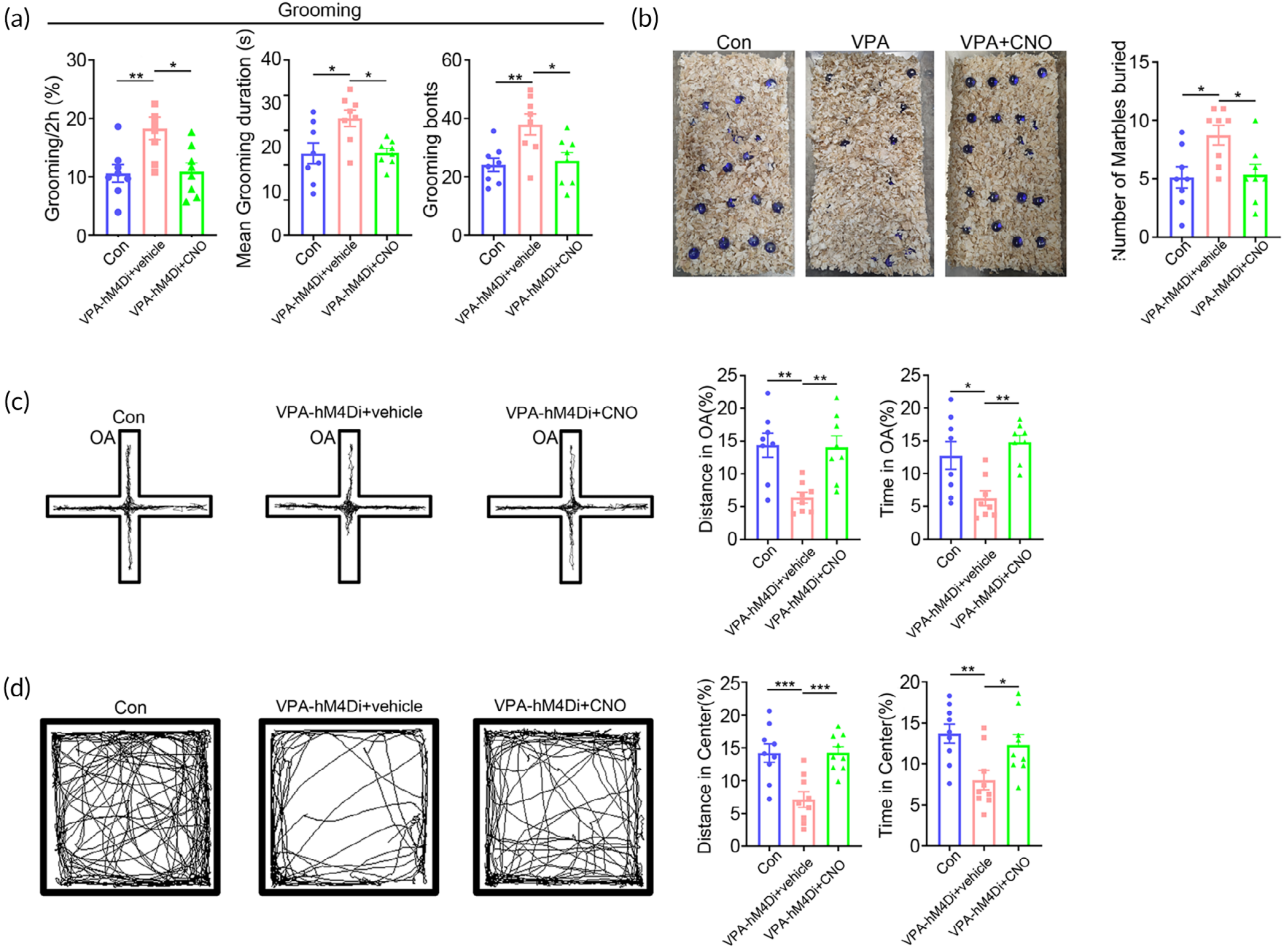
Alleviation of repetitive behaviors of ^VPA^hNPC mice by chemogenetically inhibiting human excitatory neurons. (a) Grooming test of WT mice, ^VPA‐hM4Di^hNPC mice treated with vehicle or CNO. (b) Marble burying test of WT mice, ^VPA‐hM4Di^hNPC mice treated with vehicle or CNO. Notice the decrease of repetitive behavior of ^VPA‐hM4Di^hNPC mice by CNO treatment. (c) Elevated plus maze test of WT mice, ^VPA‐hM4Di^hNPC mice treated with vehicle or CNO. (d) Open field test of WT mice, ^VPA‐hM4Di^hNPC mice treated with vehicle or CNO. Notice that CNO treatment significantly enhanced animal movement in the open arm and center field. *N* = 8 mice per group. One way ANOVA. *p < 0.05.**p < 0.01. ***p < 0.001. CNO, clozapine‐N‐oxide; Con, control chimeric mice; VPA‐hM4Di, ^VPA‐hM4Di^hNPC mice.

In terms of repetitive behaviors, CNO treatment dramatically reduced the time and frequency of self‐grooming and the numbers of marbles buried (Figure 6a,b), as compared with vehicle treatment. Further analysis showed that CNO treatment also attenuated the anxiety‐like behaviors of ^VPA‐hM4Di^hNPC mice as evidenced by the increase of moving time in the open arm (OA) of elevated cross maze and center area of open field (Figure 6c,d). These data illustrated that suppressing the human neuronal activity by CNO could alleviate most of the behavior abnormality of the VPA‐pretreated hNPC chimeric mice, supporting the idea that the ASD‐like behavior of ^VPA^hNPC mice might be caused by the overgeneration of excitatory human neurons.

### 3.4 Rescue of ASD‐like behaviors of ^VPA^hNPC mice by precisely targeted TMS

**FIGURE 7:**
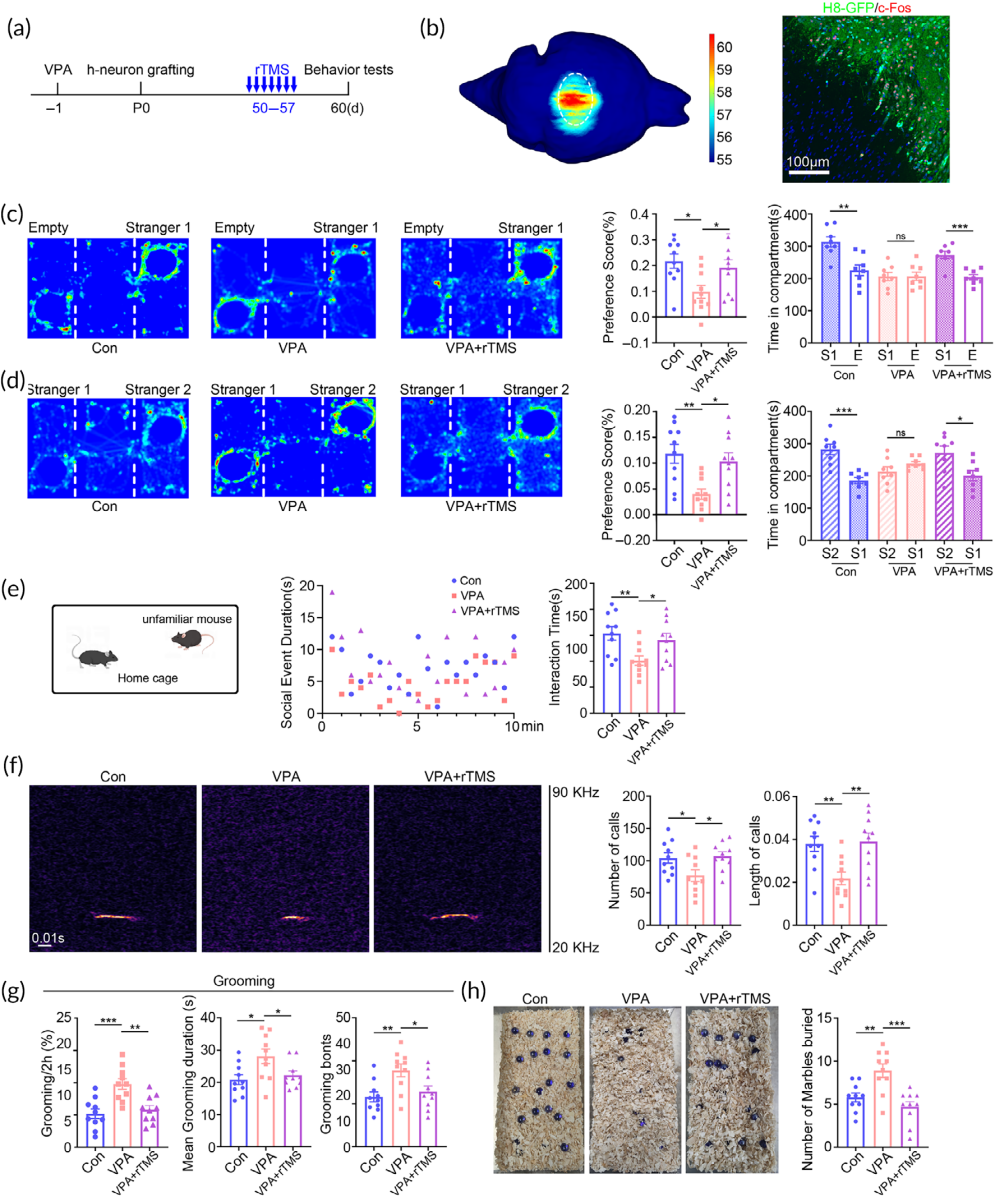
Amelioration of ASD‐like behaviors of ^VPA^hNPC mice by precisely targeted rTMS. (a) Experimental design for “b–h”. (b) Computer simulation of the rTMS‐induced electric field and c‐Fos staining following rTMS treatment. Notice the c‐Fos‐positive cells in hNPC graft but not in mouse cortex. (c, d) Three‐chamber test of WT mice, ^VPA^hNPC mice treated with or without rTMS. (e) Resident‐intruder assay of WT mice, ^VPA^hNPC mice treated with or without rTMS. (f) Ultrasonic vocalization of WT mice, ^VPA^hNPC mice treated with or without rTMS. Notice the increase of social preference, social interaction and ultrasonic communication of ^VPA^hNPC mice by precisely targeted rTMS. (g, h) Grooming test and marble burying test of WT mice, ^VPA^hNPC mice treated with or without rTMS. Notice the decrease of repetitive behavior of ^VPA^hNPC mice by rTMS treatment. *N* = 10 mice per group. One way ANOVA. *p < 0.05.**p < 0.01. ***p < 0.001. Con, control chimeric mice; rTMS, repeated transcranial magnetic stimulation; VPA, ^VPA^hNPC mice.

To modulate the activity of human neurons in a clinically appliable way, we developed a precisely targeted transcranial magnetic stimulation (TMS), which could confine magnetic stimulation to a size of approximately 0.5–2 mm^3^. We first testified the brain region specificity of this precisely targeted TMS by placing the coil on the surface of left motor cortex, and triggering right hindlimb movement by TMS stimulation (Video S1). Electromyography recording showed typical movement evoked potential in right hindlimb (but not in left hindlimb) upon TMS stimulation, illustrating the brain region specificity of this precisely targeted TMS (Figure S4). Then we stimulated the cortical area where hNPC transplantation was made, and confined magnetic field within hNPCs graft for 7 consecutive days using a low‐frequency repeated TMS (rTMS) protocol which presumably exerts inhibitory effects on neuronal activity (Figure 7a).^25^, ^26^ Computer simulation showed that the rTMS‐induced electric field overlapped well with the hNPC graft (Figure 7b, left panel). In addition, c‐Fos staining immediately following rTMS treatment revealed large number of c‐Fos positive cells in the grafting area, while very few c‐Fos positive cells could be found in adjacent mice cortex (Figure 7b, right panel). These data demonstrated the region‐specificity of this precisely targeted rTMS.

Three‐chamber assay showed that rTMS treatment significantly increased the social preference and social novelty of ^VPA^hNPC mice (Figure 7c,d). It also boosted the interacting time of ^VPA^hNPC mice with intruder mice in resident‐intruder assay (Figure 7e). Notably, rTMS treatment significantly promoted the ultrasonic emission of ^VPA^hNPC mice. The number and average duration of ultrasonic vocalization recovered to the similar levels of WT mice (Figure 7f). In addition, remarkable reduction of self‐grooming and marble burying was observed in rTMS‐treated ^VPA^hNPC mice as well (Figure 7g,h). In open‐field test and elevated plus maze test, rTMS‐treated ^VPA^hNPC mice showed no significant changes of anxiety‐like behaviors (Figure S5A,B). These data demonstrated that precisely targeting hNPCs by rTMS could rescue the core ASD syndromes of ^VPA^hNPC mice.

### 3.5 Rebalance of human neuronal differentiation and axonal projection by precisely targeted rTMS

**FIGURE 8:**
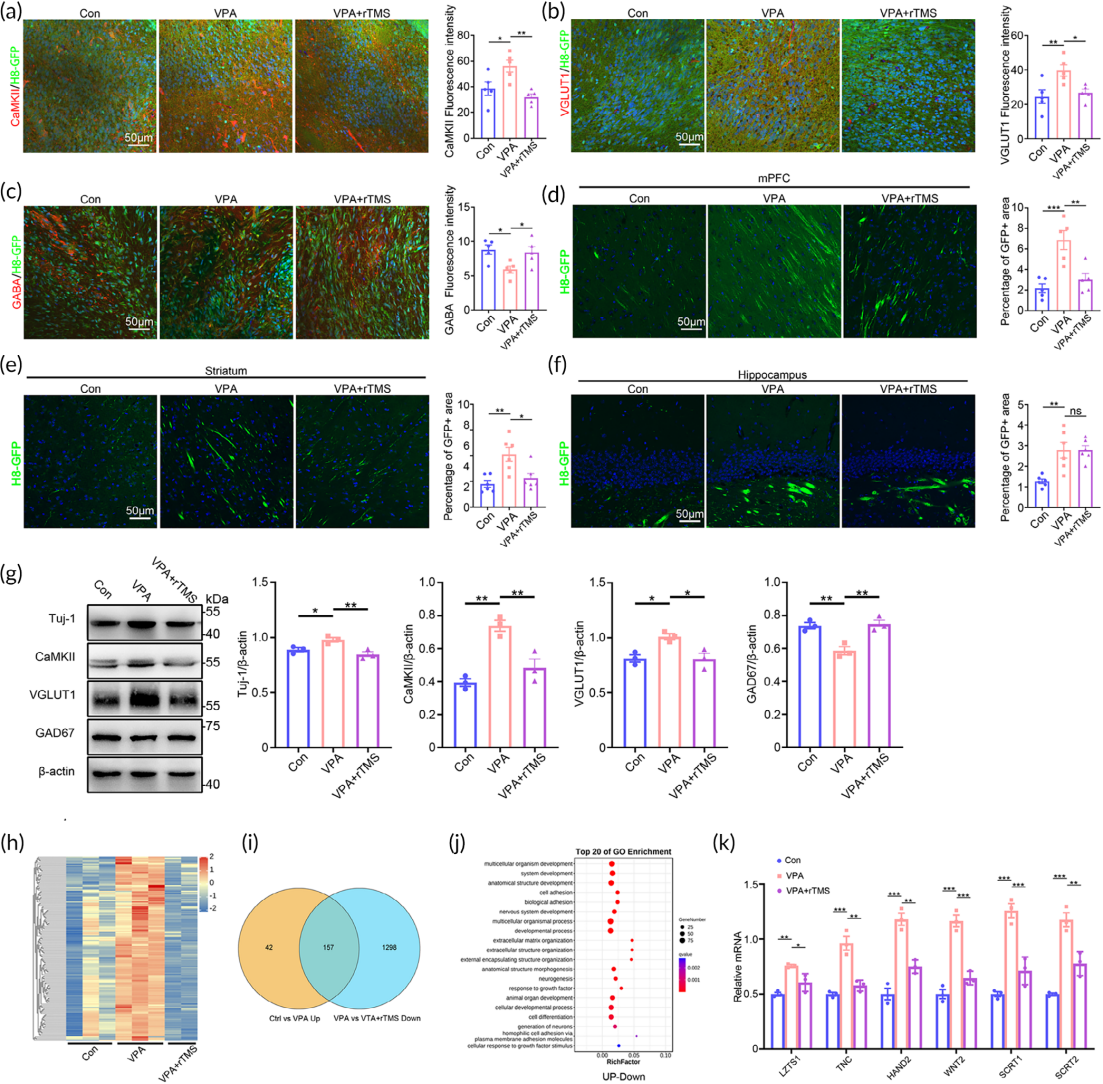
Restoration of neuronal differentiation and axonal projection of ^VPA^hNPC mice by precisely targeted rTMS. (a) Double‐immunostaining of CaMKII/H8‐GFP in WT mice, ^VPA^hNPC mice treated with or without rTMS. (b) Double‐immunostaining of Vglut1/H8‐GFP in WT mice, ^VPA^hNPC mice treated with or without rTMS. (c) Double‐immunostaining of GABA/H8‐GFP in WT mice, ^VPA^hNPC mice treated with or without rTMS. Notice the decrease of CaMKII/H8‐GFP‐positive and Vglut1/GFP‐positive cells, but the increase of GABA/H8‐GFP‐positive cells in rTMS‐treated ^VPA^hNPC mice. (d–f) GFP‐staining in the mPFC, striatum and hippocampus. Notice the decrease of human axons in the mPFC and striatum of rTMS‐treated ^VPA^hNPC mice. (g) Western‐blotting and quantification of Tuj‐1, CamKII, vGlut1 and GAD‐67 in the hNPC graft of control chimeric mice, ^VPA^hNPC mice and rTMS‐treated ^VPA^hNPC mice. (h–j) Heat‐map, Venn diagram and GO analysis of “up‐down” regulated genes in hNPCs from control chimeric mice, ^VPA^hNPC mice and rTMS‐treated ^VPA^hNPC mice. Notice the 157 “up‐down” genes and the enrichment of “neurogenesis” and “cell differentiation” in the top GO list. (k) qPCR validation of common top genes (6 out of 10) in “neurogenesis” and “cell differentiation”. *N* = 4–6 mice per group (a–g), 3 mice per group (h–k). One way ANOVA. *p < 0.05.**p < 0.01. ***p < 0.001. Con, control chimeric mice; rTMS, repeated transcranial magnetic stimulation; VPA, ^VPA^hNPC mice.

In general, rTMS is used to modulate neuronal activity by its induced electric field. Recent studies suggested that rTMS could also affect neuronal differentiation.^27^, ^28^ Given that VPA‐treated hNPCs showed abnormal neuronal differentiation, we investigated whether our rTMS protocol could alter the human neuronal differentiation of ^VPA^hNPC mice. The results showed that, in comparison with control ^VPA^hNPC mice, rTMS‐treated ^VPA^hNPC mice had significantly less CaMKII/GFP‐positive and vGlut1/GFP‐positive cells in the graft (Figure 8a,b). In contrast, rTMS‐treated ^VPA^hNPC mice showed robust increase of GABA/GFP‐positive cells in the graft (Figure 8c). Subsequently, we examined the effects of rTMS treatment on the axonal projection of human neurons, the results showed significantly fewer human axons in the mPFC and striatum of rTMS‐treated ^VPA^hNPC mice, as compared with that of control ^VPA^hNPC mice (Figure 8d,e). No changes of human axons were found in the hippocampus between rTMS‐treated ^VPA^hNPC mice and control ^VPA^hNPC mice (Figure 8f), indicating that the induced electric field might not cover all the human neurons. Western‐blotting confirmed the restoration of the protein levels of Tuj‐1, CaMKII, vGlut1 and GAD‐67 in rTMS‐treated hNPC grafts (Figure 8g). These data demonstrated that rTMS treatment could reverse the VPA‐biased excitatory/inhibitory neuronal differentiation and axonal projection.

To explore how rTMS affected the neuronal differentiation and axonal projection of hNPCs, we analyzed the transcription profiles of hNPC graft in control chimeric mice, ^VPA^hNPC mice, and rTMS‐treated ^VPA^hNPC mice by RNA‐seq. Ninety‐five genes were significantly down‐regulated in ^VPA^hNPCs and recovered in rTMS‐treated ^VPA^hNPCs, showing a “down‐up” expression pattern (Figure S6A,B). 157 genes were significantly up‐regulated in ^VPA^hNPCs and recovered in rTMS‐treated ^VPA^hNPCs, showing a “up‐down” expression pattern (Figure 8h,i). GO analysis showed that most of top 20 biological processes of “down‐up” genes were not closely relevant to the phenotype we observed (Figure S6C), while the top 20 biological processes of those “up‐down” genes enriched “neurogenesis” and “cell differentiation” (Figure 8j), which fitted well with the changes of neuronal differentiation and axonal projection we observed. We thus focused on the “up‐down” genes. The expression of top 10 genes shared by “neurogenesis” and “cell differentiation” were examined by qPCR. The results confirmed six genes in hNPC graft exhibiting the “up‐down” expression pattern following rTMS treatment (Figure 8k). Among these 6 genes, *LZTS1*,^29^, ^30^, ^31^, ^32^, ^33^ *WNT2* ^30^ and *HAND2* ^32^ are invovled in neurogenesis. *TNC* and *SCRT1/2* mediate extracellular and cell–cell interactions during axonal projection.^34^, ^35^ These data indicated that, besides modulating human neuronal activity, rTMS could also affect neuronal differentiation and long‐term gene transcription.

## 4 DISCUSSION

In the present study, we first established a humanized ASD mouse model by transplanting VPA‐pretreated hNPC into the cortex of immune‐deficient NOD‐SCID mice (designated as ^VPA^hNPC mice). ^VPA^hNPC mice exhibited social dysfunction, repetitive behaviors, excitatory‐biased human neuronal differentiation and axonal projection. Chemogenetic inhibition of human neuronal activity prevented the appearance of ASD‐like behaviors, confirming the roles of human neurons in the phenotype of ^VPA^hNPC mice. By applying a novel precisely targeted rTMS, we observed effective rescue of the core phenotypes, restoration of neuronal differentiation and of axonal projection, and even reversion of gene expression pattern in ^VPA^hNPC mice. Our data, for the first time, established an environmental factor induced humanized mouse model of ASD and demonstrated rTMS as a potential therapeutic treatment.

Humanized mice made from patient‐derived neural cells have been thought as a promising model to dissect the human‐specific mechanisms of neurodegenerative diseases such as Alzheimers disease and Huntington's disease.^15^, ^36^ So far as we know, only one paper reported humanized mice of ASD, which were made by transplanting *Shank3* ^*−/−*^ hNPCs into the lateral ventricles of mouse. The authors observed reduced axon projection and soma size of *Shank3* ^*−/−*^ human neurons as compared with that of normal human neurons.^37^ In the present study, we made chimeric mice by transplanting VPA‐pretreated hNPCs, which represents environmental factor induced ASD. To avoid the uncontrollable hNPCs migration from lateral ventricle, we transplanted VPA‐pretreated hNPCs into the somatosensory cortex of NOD‐SCID mice as described.^38^ We observed long‐term survival of human neurons and wide distribution of human axons in host brain as previously reported.^17^ More importantly, we observed human neuronal activity dependent ASD‐like behaviors in ^VPA^hNPC mice but not in control chimeric mice. Thus, ^VPA^hNPC mice could be used as a humanized model for environmental factor induced ASD. In the present study, we mainly investigated the phenotype of male ^VPA^hNPC mice, because clinical data showed that the male‐to‐female ratio of ASD patients is 3–4:1.^39^, ^40^ Whether there were similar changes in female ^VPA^hNPC mice needs to be further explored.

Most animal studies have focused on the synaptic toxicity of VPA, such as increased excitatory/inhibitory synaptic transmission ratio.^41^, ^42^ Since VPA was delivered at early pregnant stage, it is highly possible that VPA functions through affecting neuronal differentiation. Previous studies have demonstrated that different concentrations of VPA exerted different effects on the suvival, proliferation and neuronal differentiation of human embryonic stem cells.^43^, ^44^ We adopted a dose which reportedly induced neuronal differentiation of mouse neural stem cells and in human embryonic stem cells.^20^, ^45^, ^46^ Our data, for the first time, provided in‐vivo evidence that VPA pre‐treatment biased the differentiation of hNPCs towards higher excitatory/inhibitory neuron ratio, which was in line with previous observation of reduction of PV+ interneurons in the striatum of VPA‐exposed mice.^47^ Human neurons may exert their function either by themselves or by integrating into host brain.^17^ Our data that social stimulation induced c‐Fos in human neurons and chemogenetic inhibition of human neurons corrected the behavior phenotypes of ^VPA^hNPC mice supported this idea. As excitatory neurons are usually projection neurons and inhibitory neurons usually local neurons, the aberrant over‐projection we found in ^VPA^hNPC mice may be the results of over‐generated excitatory neurons. The over projection of VPA‐pretreated human neurons to mPFC and striatum may contribute to the social dysfunction and repetitive behaviors respectively, which indicate an imblance of neural circuit development in ASD patients. In addition, the changes of large‐scale gene transcription reflected the HDAC‐inhibiting function of VPA,^48^ and further indicated that overgeneration of excitatory neurons may be one of the key underpinning mechanisms for VPA‐induced ASD in human patients.

rTMS has recently been proposed as a promising therapeutic approach to mood‐defective disorders.^49^ Previous animal studies, including ours, have demonstrated its effectiveness in improving social function.^25^, ^50^ rTMS can either activates or inhibits neuronal excitation and the corresponding spreading via induced electric field. However, in most animal studies, the magnetic field covers the whole cortex, leaving the targeting area unspecific. In the present study, we adopted a newly developed 3‐coil TMS transducer which could confine the magnetic field within the hNPC graft,^51^ as evidenced by c‐fos expression in hNPC graft and coupling of motor cortex stimulation with hindlimb movement. Our stimulating protocol (1 Hz), which presumably inhibits neuronal activity, not only rescued most ASD‐like behaviors but also rebalanced the excitatory/inhibitory human neuronal differentiation in ^VPA^hNPC mice. Consistently, the gene expression pattern in ^VPA^hNPC mice was largely reversed by TMS. These data indicated that electric field not only modulate activity of mature neurons, but also influence the differentiation of hNPCs. More importantly, it implies that the time window of TMS treatment in clinic may be extended to an earlier developmental stage. Further translational study is to be performed.

## 5 CONCLUSION

Chimeric mice made from VPA‐pretreated human neural progenitors could be used as a humanized model of ASD. VPA exposure biases human neuronal differentiation and axonal projection. Precisely targeted TMS could reverse the VPA‐biased human neuronal differentiation in vivo.

## AUTHOR CONTRIBUTIONS

Y. Hou and Y. Zhao performed most of the experiments and contributed equally to this work. D. Yang contributed to ultrasonic vocalization analysis. X. Li, Z. Liu, X. Yan contribute to TMS treatment and electromyograph recording. Y. Wang, S. Wu, and X. Liu conceived and supervised the study, provided financial support, and prepared the manuscript. All authors approved the final manuscript.

## CONFLICT OF INTEREST STATEMENT

The authors declare no conflict of interest.

## Supporting information


**Figure S1.** In vitro effects of VPA on the neuronal differentiation and survival of hNPCs. (A) Double‐immunostaining of Nestin/H8‐GFP in induced hNPCs and quantification. (B) Double‐immunostaining of Tuj‐1/H8‐GFP in control and VPA‐treated hNPCs and quantification. (C) TUNEL staining in control and VPA‐treated hNPCs. *N* = 3–5 batches of cells per group. Students' *t* test. *p < 0.05.**p < 0.01. Con, control.

**Figure S2.** Cognitive behaviors of ^VPA^hNPC mice. (A) Fear conditioned memory. (B) Novel object recognition test. ^VPA^hNPC mice showed similar fear memory and novel object exploration as control chimeric mice did. *N* = 8 mice per group. One way ANOVA. *p < 0.05. WT, wild type. Con, control hNPC chimeric mice. VPA, ^VPA^hNPC mice. ns, no significance.

**Figure S3.** Effects of VPA on synaptogenesis of human neurons in vivo. (A) Double‐immunostaining of PSD‐95/H8‐GFP in hNPC grafts of control chimeric mice and ^VPA^hNPC mice. (B) Western‐blotting of PSD‐95 and Synaptophysin in hNPC grafts of control chimeric mice and ^VPA^hNPC mice. *N* = 3–4 mice per group. Students' *t* test. Con, control hNPC chimeric mice. VPA, ^VPA^hNPC mice. ns, no significance.

**Figure S4.** Effects of unilateral cortical TMS stimulation on hindlimb evoked potential. Muscle movement evoked potential induced by left motor cortex TMS stimulation, corresponding to the Video S1. Notice the movement evoked potential in right hindlimb but not in left hindlimb.

**Figure S5.** Effects of precisely targeted rTMS on the anxiety‐like behaviors of ^VPA^hNPC mice. (A) Elevated open arm maze of WT mice, and ^VPA^hNPC mice treated with or without rTMS. (B) Open field test of WT mice, and ^VPA^hNPC mice treated with or without rTMS. No significant change of anxiety‐like behaviors was found in rTMS treated ^VPA^hNPC mice. Students' *t* test. *p < 0.05. **p < 0.01. Con, control hNPC chimeric mice. VPA, ^VPA^hNPC mice. ns, no significance.

**Figure S6.** RNA‐seq of hNPCs from control chimeric mice, ^VPA^hNPC mice and rTMS‐treated ^VPA^hNPC mice. (A, B) Heatmap and Venn diagram of significantly “down‐up” regulated genes in hNPCs of control chimeric mice, ^VPA^hNPC mice and rTMS‐treated ^VPA^hNPC mice. Ninety‐five genes showed “down‐up” expression pattern. (C) Top 20 GO enriched biological processes of these significantly changed genes. Con, control hNPC chimeric mice. VPA, ^VPA^hNPC mice.

**Video S1.** Supporting Information video for Figure S2 showing the right hindlimb upon TMS stimulation on the left M1 region.

## Data Availability

The datasets used and/or analyzed during the current study are available from the corresponding author upon reasonable request.
