# Supplementary figures and images for: Amelioration of biased neuronal differentiation in humanized mouse model of valproic acid‐induced autism by precisely targeted transcranial magnetic stimulation
Source: Bioeng Transl Med. 2025 Jan 23;10(3):e10748. doi: 10.1002/btm2.10748 (PMC12079372; doi:10.1002/btm2.10748)

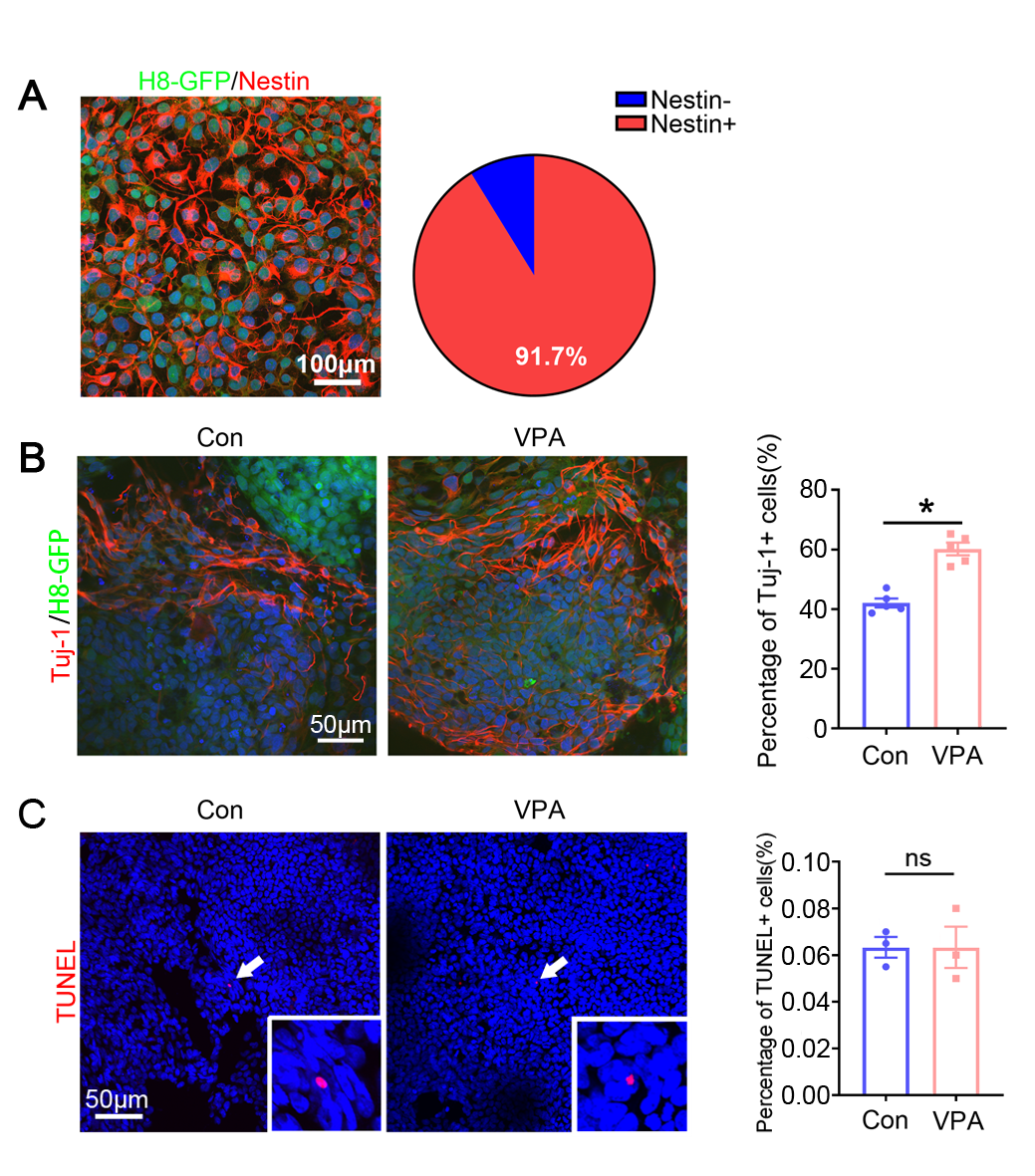

Supplement: Supplementary file 1 — Figure S1. In vitro effects of VPA on the neuronal differentiation and survival of hNPCs. (A) Double‐immunostaining of Nestin/H8‐GFP in induced hNPCs and quantification. (B) Double‐immunostaining of Tuj‐1/H8‐GFP in control and VPA‐treated hNPCs and quantification. (C) TUNEL staining in control and VPA‐treated hNPCs. N = 3–5 batches of cells per group. Students' t test. *p < 0.05.**p < 0.01. Con, control. [file BTM2-10-e10748-s004.tif]

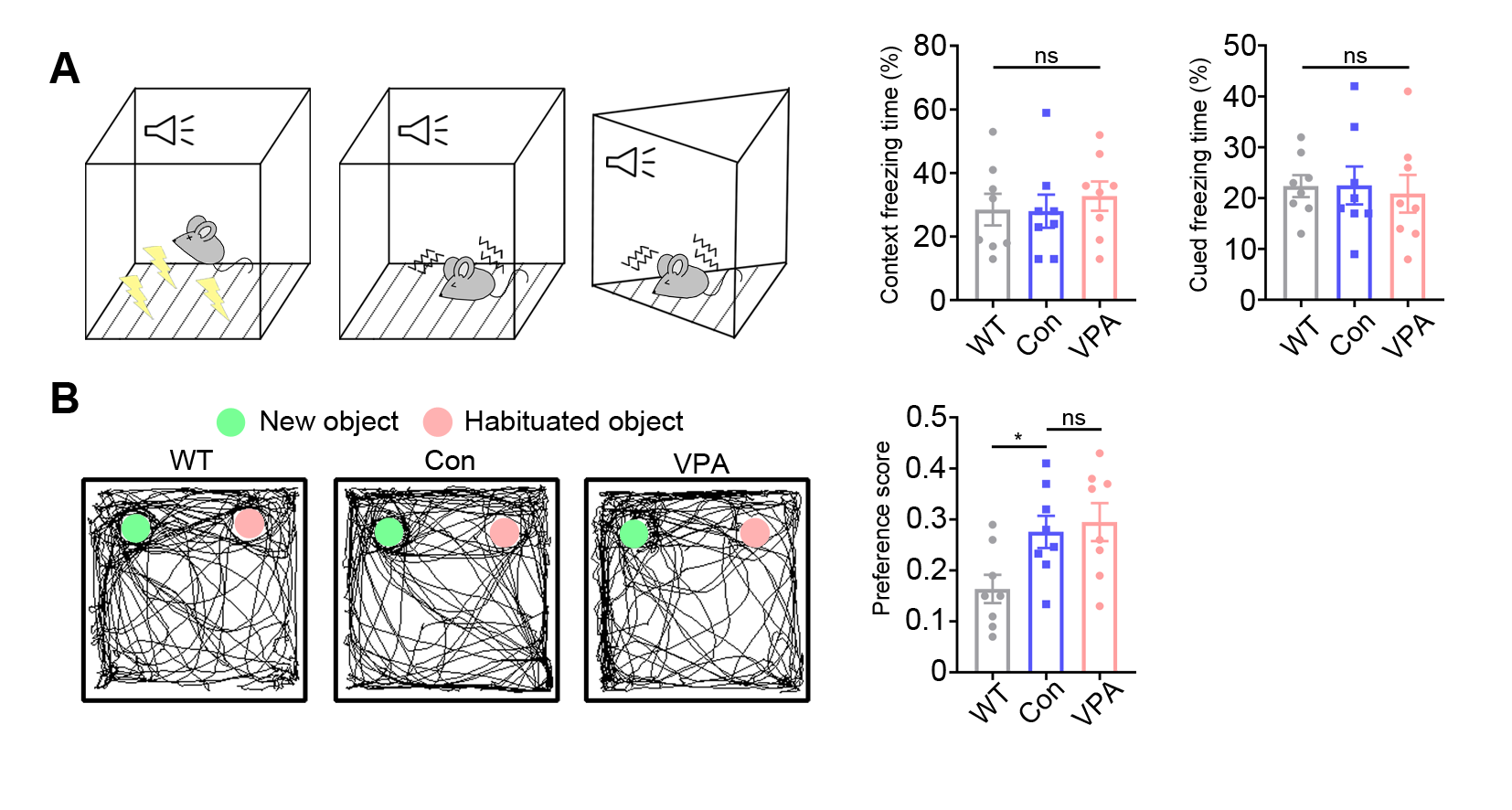

Supplement: Supplementary file 2 — Figure S2. Cognitive behaviors of VPAhNPC mice. (A) Fear conditioned memory. (B) Novel object recognition test. VPAhNPC mice showed similar fear memory and novel object exploration as control chimeric mice did. N = 8 mice per group. One way ANOVA. *p < 0.05. WT, wild type. Con, control hNPC chimeric mice. VPA, VPAhNPC mice. ns, no significance. [file BTM2-10-e10748-s007.tif]

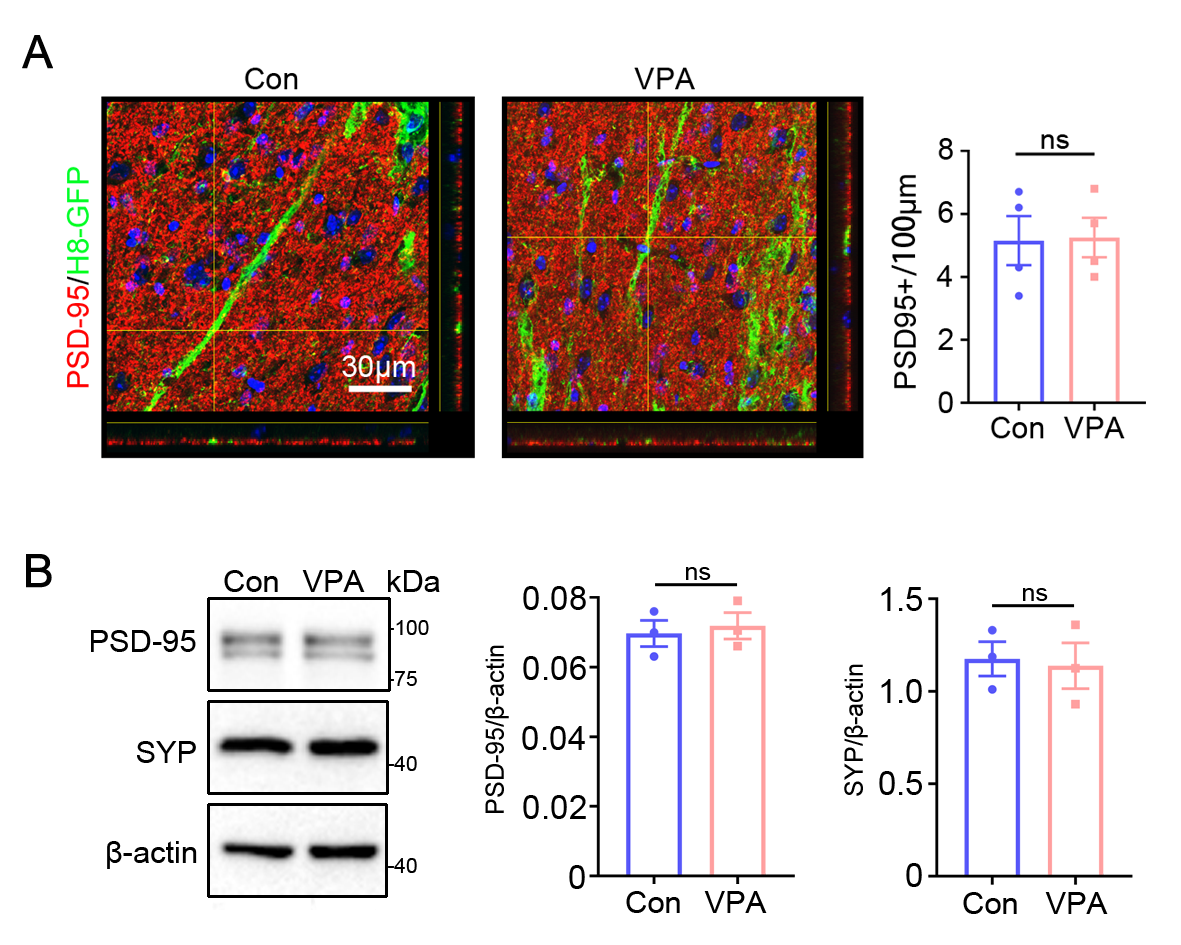

Supplement: Supplementary file 3 — Figure S3. Effects of VPA on synaptogenesis of human neurons in vivo. (A) Double‐immunostaining of PSD‐95/H8‐GFP in hNPC grafts of control chimeric mice and VPAhNPC mice. (B) Western‐blotting of PSD‐95 and Synaptophysin in hNPC grafts of control chimeric mice and VPAhNPC mice. N = 3–4 mice per group. Students' t test. Con, control hNPC chimeric mice. VPA, VPAhNPC mice. ns, no significance. [file BTM2-10-e10748-s001.tif]

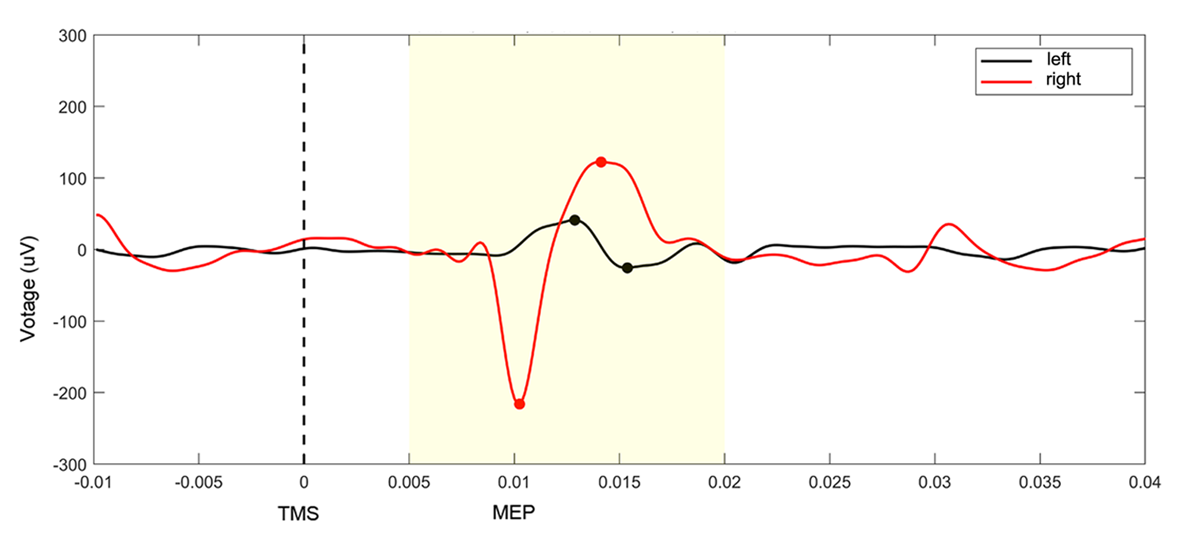

Supplement: Supplementary file 4 — Figure S4. Effects of unilateral cortical TMS stimulation on hindlimb evoked potential. Muscle movement evoked potential induced by left motor cortex TMS stimulation, corresponding to the Video S1. Notice the movement evoked potential in right hindlimb but not in left hindlimb. [file BTM2-10-e10748-s006.tif]

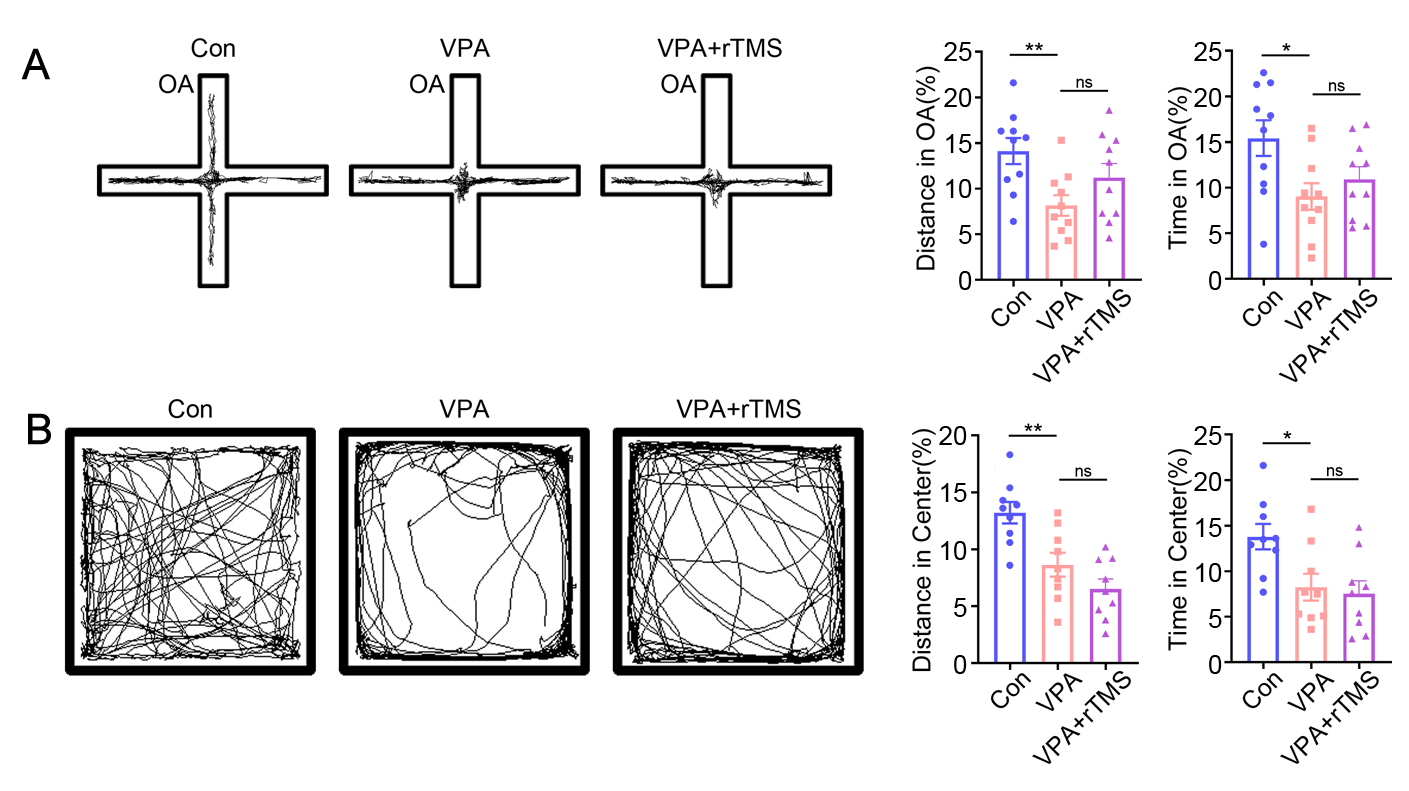

Supplement: Supplementary file 5 — Figure S5. Effects of precisely targeted rTMS on the anxiety‐like behaviors of VPAhNPC mice. (A) Elevated open arm maze of WT mice, and VPAhNPC mice treated with or without rTMS. (B) Open field test of WT mice, and VPAhNPC mice treated with or without rTMS. No significant change of anxiety‐like behaviors was found in rTMS treated VPAhNPC mice. Students' t test. *p < 0.05. **p < 0.01. Con, control hNPC chimeric mice. VPA, VPAhNPC mice. ns, no significance. [file BTM2-10-e10748-s002.tif]

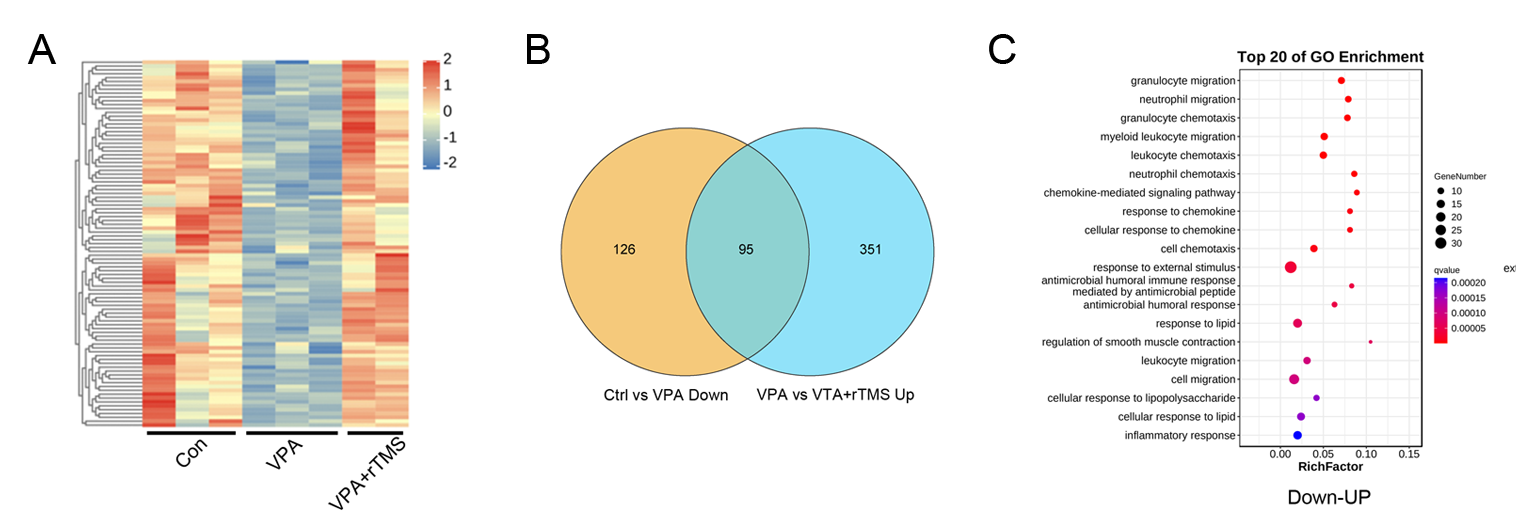

Supplement: Supplementary file 6 — Figure S6. RNA‐seq of hNPCs from control chimeric mice, VPAhNPC mice and rTMS‐treated VPAhNPC mice. (A, B) Heatmap and Venn diagram of significantly “down‐up” regulated genes in hNPCs of control chimeric mice, VPAhNPC mice and rTMS‐treated VPAhNPC mice. Ninety‐five genes showed “down‐up” expression pattern. (C) Top 20 GO enriched biological processes of these significantly changed genes. Con, control hNPC chimeric mice. VPA, VPAhNPC mice. [file BTM2-10-e10748-s003.tif]
